# Characterization of Pathogen Airborne Inoculum Density by Information Theoretic Analysis of Spore Trap Time Series Data

**DOI:** 10.3390/e22121343

**Published:** 2020-11-27

**Authors:** Robin A. Choudhury, Neil McRoberts

**Affiliations:** 1 School of Earth, Environmental, and Marine Sciences, University of Texas, Rio Grande Valley, Edinburg, TX 78541, USA; robchoudhury@gmail.com; 2 Quantitative Biology and Epidemiology Group, Plant Pathology Department, University of California, Davis, Davis, CA 95616, USA

**Keywords:** time series, entropy, average mutual information, stochastic processes, deterministic dynamics

## Abstract

In a previous study, air sampling using vortex air samplers combined with species-specific amplification of pathogen DNA was carried out over two years in four or five locations in the Salinas Valley of California. The resulting time series data for the abundance of pathogen DNA trapped per day displayed complex dynamics with features of both deterministic (chaotic) and stochastic uncertainty. Methods of nonlinear time series analysis developed for the reconstruction of low dimensional attractors provided new insights into the complexity of pathogen abundance data. In particular, the analyses suggested that the length of time series data that it is practical or cost-effective to collect may limit the ability to definitively classify the uncertainty in the data. Over the two years of the study, five location/year combinations were classified as having stochastic linear dynamics and four were not. Calculation of entropy values for either the number of pathogen DNA copies or for a binary string indicating whether the pathogen abundance data were increasing revealed (1) some robust differences in the dynamics between seasons that were not obvious in the time series data themselves and (2) that the series were almost all at their theoretical maximum entropy value when considered from the simple perspective of whether instantaneous change along the sequence was positive.

## 1. Introduction

“*We now have to look at apparently random time series of data, be they from the stock market, or currency exchanges, or in ecology and ask are we seeing “random walks down Wall street” or deterministic chaos, or, often more likely, some mixture of the two*.”

—Sir Robert May [1]

The study of disease dynamics in plant pathology has been dominated by analysis of situations where disease increases monotonically within single growing seasons or over several seasons [2]. Reflecting this focus, the literature on the use of monotonic growth curve models or, more recently, compartment models consisting of linked differential equations is extensive and the methodology is well developed. In contrast, the literature on how to handle long, oscillating data series for plant pathogen populations is rather thin, with only isolated case studies [3,4,5,6,7] employing a range of statistical approaches. To date, there has been no concerted effort in botanical epidemiology to establish general properties of time series data associated with pathogen populations or disease intensity. This is due in part, no doubt, to the fact that time series methods have been considered relevant mostly for multi-season contexts, and multi-season datasets are scarce in plant pathology. However, with the advent of molecular probes for studying the airborne inoculum of plant pathogens, it has become much easier to capture time series data within single growing seasons [5,6,8].

Developments in technology for monitoring airborne inoculum of target species offer a promise of methodological advance to epidemiologists with an interest in creating evidence-based, within-season decision rules for disease management in crops. Given such potential applications for spore traps and quantification of target nucleic acid sequences, it is important that efforts are made to develop an analytical approach which takes into account the relevant statistical properties of the data that these monitoring methods generate. What can experimenters expect to see when they collect such data? What types of dynamical behavior are likely to be apparent, and how should the results be interpreted in relation to the use of the data in disease management?

The work we report here falls into the broad theme on decision-making that runs through several of the contributions to this Special Issue of Entropy. In the case of the current work, our effort is aimed more at understanding the basic properties of the data than in deriving decision rules from them. The work is motivated by our belief that it is important to be aware of any informational limitations inherent in the data, so that efforts to use air sampling as a means of forecasting interventions occur with realistic expectations. The work is intended to be an initial contribution to the literature; one from which we hope a range of further investigations covering a wider range of pathogen systems will develop.

As already noted, airborne concentrations of pathogen inoculum have been monitored using vortex (spinning rod) air samplers combined with species-specific quantitative polymerase chain reaction (qPCR) in a number of situations. In some cases, the approach has already been used commercially for disease management. Carisse and colleagues were pioneers, developing one of the first examples in commercial agriculture; in their case, to manage fungicide applications to control Botrytis leaf blight in onion in Quebec, Canada [5,9,10]. Their work (along with characterization of effective fungicide regimes and conducive weather conditions) helped to improve monitoring and to reduce disease outbreaks.

The use of spore traps linked with qPCR assays has been developed successfully for disease monitoring in several other pathosystems, including monitoring for early season inoculum for grape powdery mildew [11], where mitigating early season inoculum can reduce yield losses in susceptible varieties. These studies show that managing disease based on the binary presence or absence of pathogen primary inoculum can be quite successful, since what is needed in that situation is to detect the first occurrence of pathogen activity at the start of the growing season. The use of these systems for mitigating the impacts from secondary inoculum is more challenging.

Spinach downy mildew, caused by the obligate oomycete pathogen *Peronospora effusa*, is the most important threat to spinach production worldwide. Choudhury et al. [6] examined several sets of qPCR-based spore trap data collected from the Salinas Valley in California. The resulting time series were analyzed by fitting statistical models to characterize both trend and periodicity. While the approach was successful in producing a description of the observed dynamics, and in linking important statistical features to plausible biological mechanisms, it offered little in the way of general understanding of inoculum dynamics. Analyses of the coefficients of prediction and the Lyapunov exponents of the time series suggested that the datasets were quasi-chaotic. Further analyses of this example dataset could reveal general dynamics of airborne inoculum for plant pathogens.

Recent developments in time series analysis [12] based on information-theoretic quantities offer some promise in being able to extract more generic properties from the available data. Our objectives in this paper are to revisit the data originally studied by Choudhury et al. [6] and to apply the methods suggested by Huffaker et al. [12] in order to describe the dynamics of pathogen airborne inoculum in information theoretic terms. The analyses also place our data from botanical epidemiology in the wider context of the analysis of dynamical systems allowing interdisciplinary comparison. Our primary intended audience is plant pathologists and epidemiologists who might be interested in an introduction to these topics. For that reason, our approach is somewhat pedagogical and does not delve deeply into the underlying technical details.

## 2. Materials and Methods 

### 2.1. Data Collection

Airborne inoculum of *P. effusa* was sampled at four locations in the Salinas Valley of California in 2013 and 2014 using vortex air samplers constructed by Dr. Walt Mahaffee (USDA-ARS Corvallis, Corvallis, OR, USA) and operated by Dr. Steven Klosterman (USDA-ARS Salinas, Salinas, CA, USA). The presence of the inoculum and quantification were achieved using qPCR amplification of a species-specific DNA sequence in the total DNA extract from the sampler rods. Details of the sampling procedure, qPCR primers, reaction conditions, and translation of the qPCR cycle threshold number to daily pathogen DNA copy number are described in Klosterman et al. [8].

### 2.2. Data Preparation

Samples were recovered from the air samplers on an irregular sampling interval of two or three days depending on the availability of technical staff. In the original 2016 study [6], we accommodated the irregular sampling interval by fitting a flexible sine function to the observations, having first removed any temporal trend by linear regression. In the current work, in order to utilize nonlinear methods incorporating information quantities, we interpolated the raw data to produce time series with a regular time step of one day. All nine data series were processed in the same way so that we could compare their statistical properties directly. The interpolation was achieved by linear averaging between the measured data points. The interpolation method will have the effect of smoothing the data to some extent, and the interpretation of the results takes that into account. We avoid overinterpretation of fine-grain aspects of the analyzed series and focus on the major dynamic features that are unlikely to be strongly influenced by the interpolation.

### 2.3. Basic Time Series Analysis

After interpolation of the data to a daily time step, each of the nine time series consisted of 129 observations of the estimated target DNA copy number of *P. effusa* trapped over the preceding 24 h period. The nine time series were first inspected for evidence of an overall trend in copy number with time. Increasing trends were detected in 7 of the 9 series, and the series were tagged accordingly to indicate their status. Irrespective of whether or not the initial inspection suggested a trend to be present, in order to standardize the pretreatment of the data, a simple linear regression with time (i.e., data point in sequence, *t* = 1, 2, 3,... 129) was fitted to the natural logarithm of the estimated copy number. The residuals from the regression were then exponentiated to produce the detrended series that were subsequently used analysis. In what follows, we refer to these series as *N_t_*, indicating the (detrended) copy number on day *t*. When corresponding log-transformed values are analyzed, they are denoted *n_t_*.

For each series, we obtained the autocorrelation function (ACF), the partial autocorrelation function (PACF), and the phase plot of the log-transformed series with *n_t+1_* = ln(*N_t_*_+1_) on the ordinate and *n_t_* = ln(*N_t_*) on the abscissa. The PACF differs from the standard autocorrelation function in that it considers only the direct effect of observations at one point in the series on observations separated by lag *τ*, indirect effects, operating through the interposing points in the series that are removed.

### 2.4. Nonlinear Time Series Analysis

To characterize the time series in terms of nonlinear dynamics, we followed an approach suggested by Huffaker et al. [12] and by Kantz and Schreiber [13]. The various quantities estimated for each series were obtained using functions provided in the R packages “*nonlinearTseries*” [14], “*TseriesChaos*” [15], or “*TseriesEntropy*” [16]. Additional calculations to obtain empirical entropy values used the package “*entropy*” [17] or were coded directly in R. As with many other aspects of applied data analysis, for several of the steps in a nonlinear time series analysis, there is no single method that is guaranteed to provide optimal results under every circumstance. For many of the procedures, there are no formal test statistics to indicate that a “significant” result has been obtained; we followed the approaches suggested in the references. We provide R code and data necessary to replicate a full set of analyses for one of the 9 time series analyzed in the repository at the following URL: https://github.com/robchoudhury/spore_trap_information_theory. The R code is provided as is, and we offer no guarantee that it will work when adapted to other data sets.

#### 2.4.1. Surrogate Testing for Nonlinear Dependence

Since nonlinear analysis (NLTS) can be time-consuming, an initial step should be to test for lack of linear dependence in the observed data. An agreed approach for performing this is to perform surrogate tests [12,14]. Different versions of the surrogate test are implemented in *nonlinearTseries* and *TseriesChaos*. The basic idea in both cases is to construct an empirical hypothesis test by resampling from the observed data, with the test statistic being a suitable property of the data that will hold under linear dependence but not otherwise. One of the simplest approaches, the one implemented in *nonlinearTseries*, relies on the idea that a Gaussian linear process will show time reversibility. Randomized permutations are obtained using a method in which the phases of the Fourier transform of the observed data are randomized. A two-sided hypothesis test is implemented to examine whether there is evidence that the value calculated from the observed data differs from the set of surrogates generated in the data resampling routine. We set the “significance level” option at 0.02, which results in the observed data being treated as one observation in a set of 100, with the two-sided test examining whether the observed data are in the *p* = 0.02 upper or lower tail of the sample. The supplied function includes a built-in diagnostic plot of the resampling test, but we implemented our own diagnostic graphical representation of the outcome for the test.

*TseriesEntropy* implements a more complex surrogate testing procedure. First, the best-fitting linear autoregressive (AR) model is selected on the basis of the Akaike Information Criterion (AIC). The residuals of the best AR model are resampled (with replacement). For each resampled series, a metric entropy measure (the Bhattacharya–Matusita–Hellinger measure, *S_p_*) [18] is calculated at different lags. Based on the relevant properties of the resampled data, the 95% confidence band for *S_p_* can be calculated and the values for the observed series are compared with the confidence band. If *S_p_* for the observed series falls outside the band, the series can be considered to show nonlinear as opposed to linear dependence at the relevant lags. The entropy-based approach in *TseriesEntropy* is computationally more demanding than the expectation-based approach in *nonlinearTseries*. In the initial work, we examined both approaches. The results reported here are for the time-reversibility approach implemented in *nonlinearTseries*. The code supplied in the Supplemental Materials includes an example of the regression-based approach, deactivated by comment markers.

#### 2.4.2. Characterizing Nonlinear Properties

Assuming that the surrogate tests indicate sufficient reason to proceed with NLTS, characterization of the dynamics in terms of their tendency to chaotic versus stochastic uncertainty is an important component of the ensuing effort. Following the pioneering work of Takens [19], one widely accepted approach to NLTS proceeds by attempting to reconstruct important features of the complete (and only partially observed) phase space of the whole system, using the methods of time delay embedding to characterize the time series of a single observed component of the system.

In the current context, where the ultimate motivation is the hope of using similar series in disease management, the capacity to reconstruct the phase portrait of the whole system is of secondary importance to characterizing the dynamics of the observed series. However in this initial study the focus is on understanding the dynamics rather than immediate practical application, and the time delay embedding approach may be valuable because the features of the dynamics it reveals are informative.

Three properties of the series are important in NLTS, these being (i) the average mutual information (AMI), *I*(*N_t_*,; *N_t_*_-*τ*_), of the time series data at successive lags, *τ* = 0, 1, 2, … *τ*_max_; (ii) the Theiler Window, *tw*; and (iii) the embedding dimension, *m*.

##### The AMI Function

The AMI function is calculated by binning observations and by calculating the mutual information obtained about observation *N_t_* being in the *i*th bin from knowing observation *N_t_*_-*τ*_ is in the *j*th bin. The results are averaged over all of the available data to produce the average mutual information. A graphical plot of *I*(*N_t_*,; *N_t_*_-*τ*_) against lag, *τ* = 0, 1, 2, … *τ*_max_ produces an information-theoretic analogue of the ACF plot, but one in which the AMI’s general measure of lagged association, as opposed to the linear lagged dependence captured by ACF, is visualized. The first minimum, or the first occurrence of a value below an empirical threshold, of the AMI function is taken to be an indication of the embedding time delay, *d*, of the series, since this value indicates a time lag at which observations have, in a general sense, low correlation.

##### The Theiler Window (*tw*)

The Theiler Window [20,21] is used to define the minimum separation along the time series that two points must have in order to be included in procedures used to find the embedding dimension, *m* (see below). Theiler’s review [21] gives a detailed and technical account of the issues and the various approaches suggested (up to that time) for finding the embedding dimension.

For long time series, both *TseriesChaos* and *nonlinearTseries* offer functions to generate a space-time plot [22] from which *tw* can be selected by choosing a value at which there is a low probability of points being close in the phase space for a given time lag separation. For short time series, such as what we have in the present study, the space-time plot approach may not give usable results and other options may be needed; this was the case with our datasets which consist of 129 observations.

As an easily obtained first approximation, Huffaker et al. [12] suggested using the first minimum of the standard autocorrelation function (ACF). Since ACF is a linear function, there are risks in using it to estimate correlation structure of nonlinear data [23]; indeed, this issue was one of the motivations for Theiler’s review [21] of methods for identifying the dimensionality of nonlinear attractors. The problem, in general, appears to be that nonlinear correlation may occur at a larger lag separation that would be suggested by the ACF.

In the current case, lacking a reasonable alternative, we opted for a trial-and-error approach. With both the AMI function and the ACF available, we had estimates of both general association and linear correlation with lag, while the original time series and the corresponding phase plots also help to indicate suitable values of *tw*. For each series, we started with the value suggested by the first minimum of the ACF, noting also whether this lag separation was longer or shorter than the value suggested by the AMI. Where the AMI reached its first minimum at longer lag than the ACF, we used a range of estimates for *tw* and examined the effect of changing *tw* on the estimated embedding dimension, *m*.

##### The Embedding Dimension, *m*

Options for estimating, *m*, are either the method of False Nearest Neighbors (FNN) offered in *TseriesChaos* (Huffaker et al. [12] pp. 67–69) or Cao’s [24] algorithm implemented in *nonlinearTseries*. Briefly, the motivation for the FNN approach comes from the idea that (in the current case), the observed time series of pathogen DNA copies represent only one dimension of a higher-order dynamical system. We can think of the observed series as representing the whole higher-order dynamical system projected onto a single dimension. With this perspective, points that appear close to one another may actually be widely separated in the full dimensional space of the dynamical system. The idea of FNN computation is to select a subset of points within a given “radius” of each other but separated by at least the value of *tw* and to track whether they remain as neighbors as the dimensionality of the assumed attractor is incrementally increased. If the proportion of FNN is plotted against the number of dimensions, *m*, the first value of *m* at which the proportion of FNN is minimized provides an estimate of the embedding dimension.

In the approach suggested by Cao [24], the embedding dimension is identified by calculating a pair of functions, referred to as *E*1(*m*) and *E*2(*m*), of putative values for the embedding dimension, *m*. Note that Cao’s original notation used d in place of *m*. Cao’s method starts by calculating an overall Euclidean distance measure between pairs of points on time delay vectors for successively larger assumed values of *m*. Function *E*1(*m*) calculates the ratio of the distance measure at successive pairs of values, (*m*+1, *m*). Cao’s insight was that this ratio stabilizes close to 1 if the data are generated by an attractor. The second function, *E*2(*m*), focuses on the distance between only the nearest neighbors in the time delay vectors and operates on the distance measure based only on those. As with *E*1(*m*), the function returns the ratio between successive pairs (*m*+1, *m*). If the data are generated by a deterministic attractor, *E*2(*m*) has the property that, at some value m*, *E*2(m*)! = 1, whereas if the data are generated by a process dominated by stochastic noise, *E*2(*m*) ≅ 1, ∀*m*. Thus, in addition to providing an estimate of the relevant embedding dimension, Cao’s method offers the advantage over the FNN approach of providing an indication of whether the data-generating process is characterized by deterministic or stochastic uncertainty.

### 2.5. Additional Entropy Measures

In addition to the characterization of the dynamics provided by the time-delay-embedding approach, we calculated two empirical entropy values to help in understanding the uncertainty in the data for airborne pathogen DNA. The first approach worked directly on the DNA copy number time series (following detrending if necessary, see above). The entropy () function from the R package *entropy* was used to calculate empirical estimates of the entropy in the data at each time point by iteratively adding the datum for each time point to the entropy calculation. Calculation using this approach starts by constructing a binning structure for the data and then by estimating the entropy based on the frequencies of observation in each bin. We started the iterative process at the 10th time point, so that the first estimate of entropy was based on the first 10 observations of each series. The calculation then proceeded as just outlined, with the second estimate being based on the first 11 data points and so on. The maximum likelihood option for the entropy function was used throughout.

As a second approach to characterize uncertainty in the time series data in relation to decision making, we first transformed each series into a binary string of length (*t_max_*-1). First differences between successive pairs of values were calculated, and if the resulting difference was greater than 0 (indicating *N_t_*_+1_ > *N_t_*), then 1 was entered for the corresponding value of the string; *N_t_*_+1_ ≤ *N_t_* resulted in 0. The calculation then proceeded along similar lines to those outlined for the entropy of the copy number, iteratively increasing the size of the dataset by one time point and calculating a new entropy value. In the current case, at each time point, we calculated the proportion of the data that were 1s and then used Shannon’s equation for expected information to give an entropy value in bits for the string at each time point (including all data up to that time). The calculation was coded directly in R. We initiated the calculation with the first two observations and then iterated the calculation one time point at a time.

### 2.6. Linear Autoregressive Models

In discussing the analysis of time series data for biological populations, Royama [25] noted that for autoregressive (AR) models where an instantaneous growth rate is modeled as a function of lagged population sizes, there is a qualitative difference in the types of behavior that a second-order lag model can display compared with a first-order model. Further, given the capacity for second-order linear models to generate quite complex oscillatory patterns, even when completely deterministic, Royama [25] suggested they could be expected to approximate the behavior of simple nonlinear models. Since the main aim of our investigation is to look at the utility of nonlinear methods, the linear AR models included here were fitted for the purpose of illustrating the extent to which a linear model can account for the observed behavior of the data collected from air samplers.

We followed a conceptual approach that draws on the work of Royama [25] and Turchin [26] in fitting the AR linear models. The process starts with the log-transformed (*log_e_*) time series, denoted *n_t_*. The instantaneous log growth rate *R_t_* is defined as *n_t_*_+1_ − *n_t_*, and the estimated linear AR model is then

*R_t_* = a_0_ + a_1_*n_t_* + a_2_*n_t_*_-*τ*_+ *ε*, *τ* = 1, 2, ... *τ*_max_ (1)

in which a_0_, a_1_, etc. are parameters to be estimated; ε is an error term; and *τ* is an index indicating lag dependence. Selection of the order of lag dependence (i.e., the value *τ*_max_) to use in fitting the AR models in each case was guided by the estimates of ACF and AMI functions (see Section 2.4.2 above). Parameter estimation was achieved by the standard least-squares approach implemented in the lm () function in the R base statistics package. For the selected model in each case, we noted the percent variance accounted for by the model in form of the standard adjusted-R^2^ and a coefficient of prediction similar to the one proposed by Turchin [26]. The coefficient was obtained as follows: We fitted a model consisting of only the mean value of the dependent variable and captured the residual sum of squares, (RSS_mn_). Next, we calculated 1−(RSS_mod_/RSS_mn_), in which RSS_mod_ is the residual sum of squares from the selected model. When RSS_mod_ > RSS_mn_, the coefficient has a negative value and indicates that the model fits noise. Values approaching 1 occur when the observed series has a pattern of oscillations that can be captured reasonably well in simple autoregressive models. Finally, values in the region of 0 indicate that the series is dominated by noise and, possibly, too short and complex to be characterized well.

## 3. Results

### 3.1. Time Series Properties and Nonlinearity

**Figure 1:**
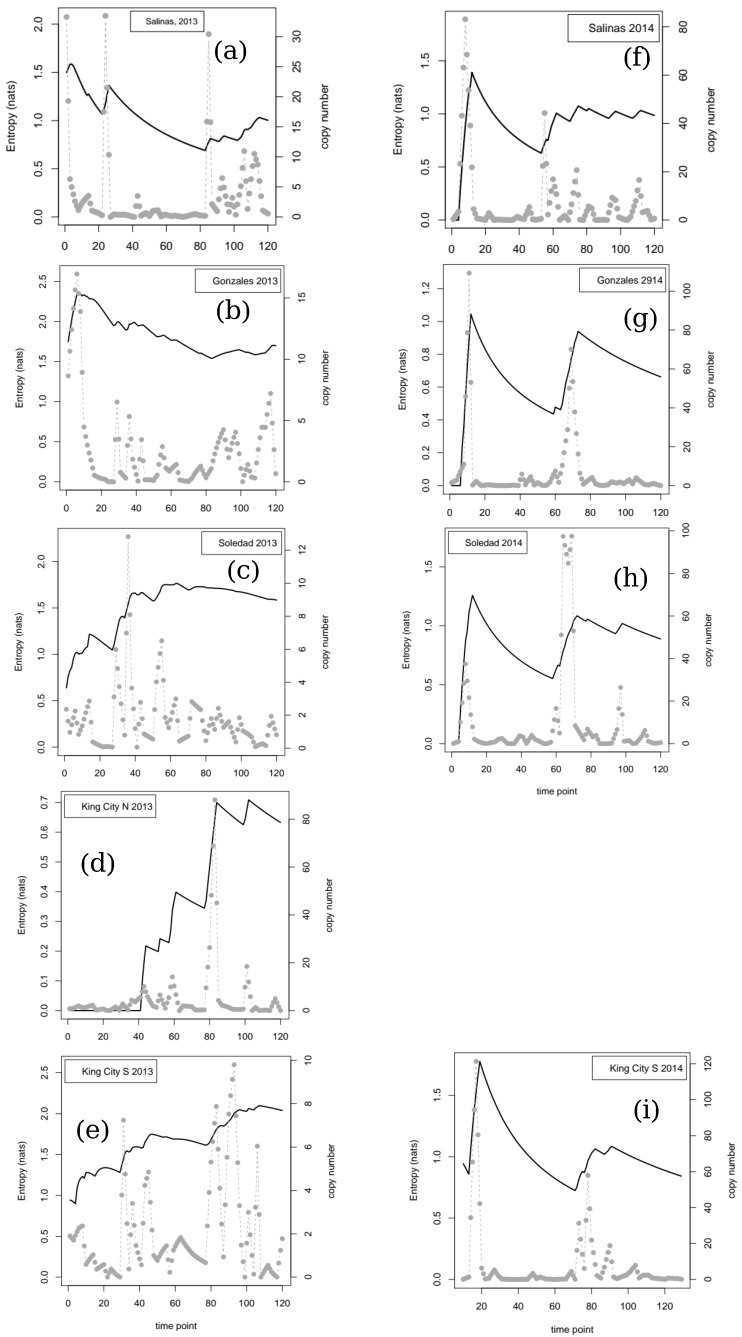
Detrended daily pathogen DNA copy number trapped (right axis scale) and cumulative entropy (nats, left axis scale) in the copy number series for 9 location/year combinations in which vortex air samplers were used to sample for the presence of DNA from the downy mildew pathogen of spinach, *P. effuse*, in the Salinas Valley of California: (**a**–**e**), 2013; (**f**–**i**), 2014. The King City, North location was sampled only in 2013.

**Figure 2:**
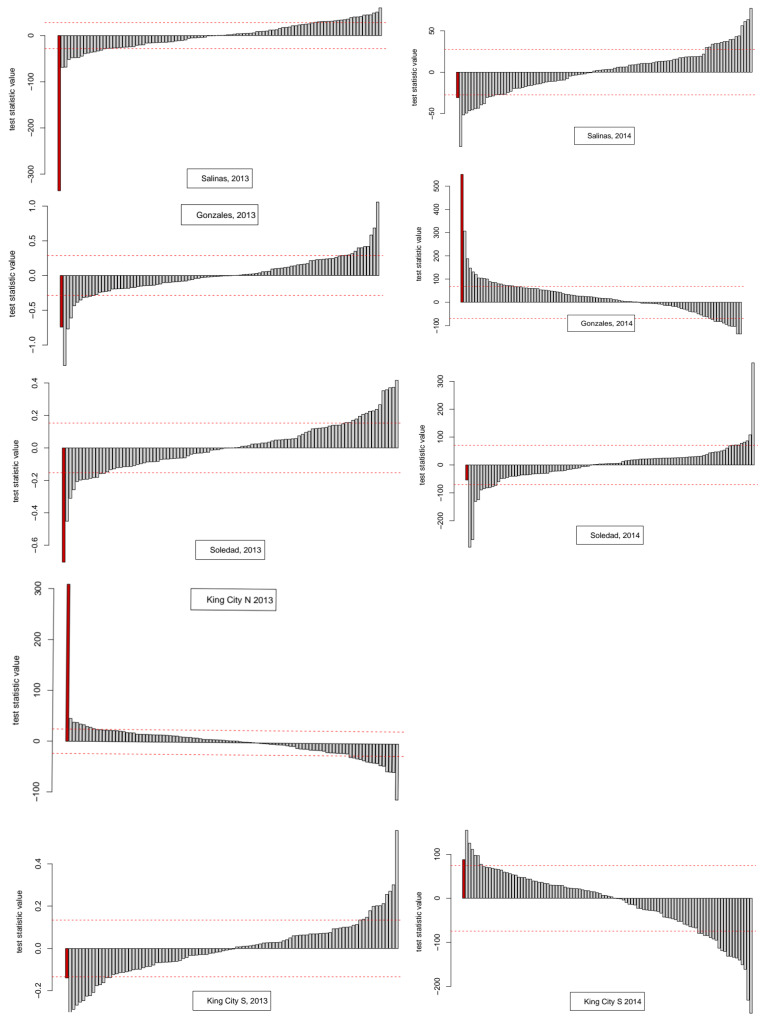
Results from surrogate tests (i.e., bootstrap data resampling) to assess the compatibility of spore trap data giving daily pathogen DNA copy numbers with time reversibility: the initial bar (in red) in each graph is the test statistic calculated for the original data. The remaining bars are the values calculated for bootstrap resamples of the data constructed in such a way to break any temporal autocorrelation in the original data. The dashed horizontal lines show the standard deviation of the surrogates above and below zero. Four of the nine series fail the two-sided hypothesis test for compatibility with time reversibility (i.e., stochastic linearity). Further details are given in the main text.

Time series graphs for the nine series of spore trap DNA copy number data are shown in Figure 1. Two of the nine series did not require detrending prior to analysis, these being King City South, 2014 and Gonzales, 2014. The results of testing for evidence of nonlinear dependence using Cao’s method are shown in Table 1 along with other summary parameters of interest for the nine series. For four series—Salinas 2013, Soledad 2013, King City North 2013, and Gonzales 2014—the surrogate (bootstrap) test led to rejection of the null hypothesis that the data were compatible with a stochastic linear (i.e., time-reversible) process. The output from the bootstrap analysis for each series is shown in Figure 2.

**Figure A1:**
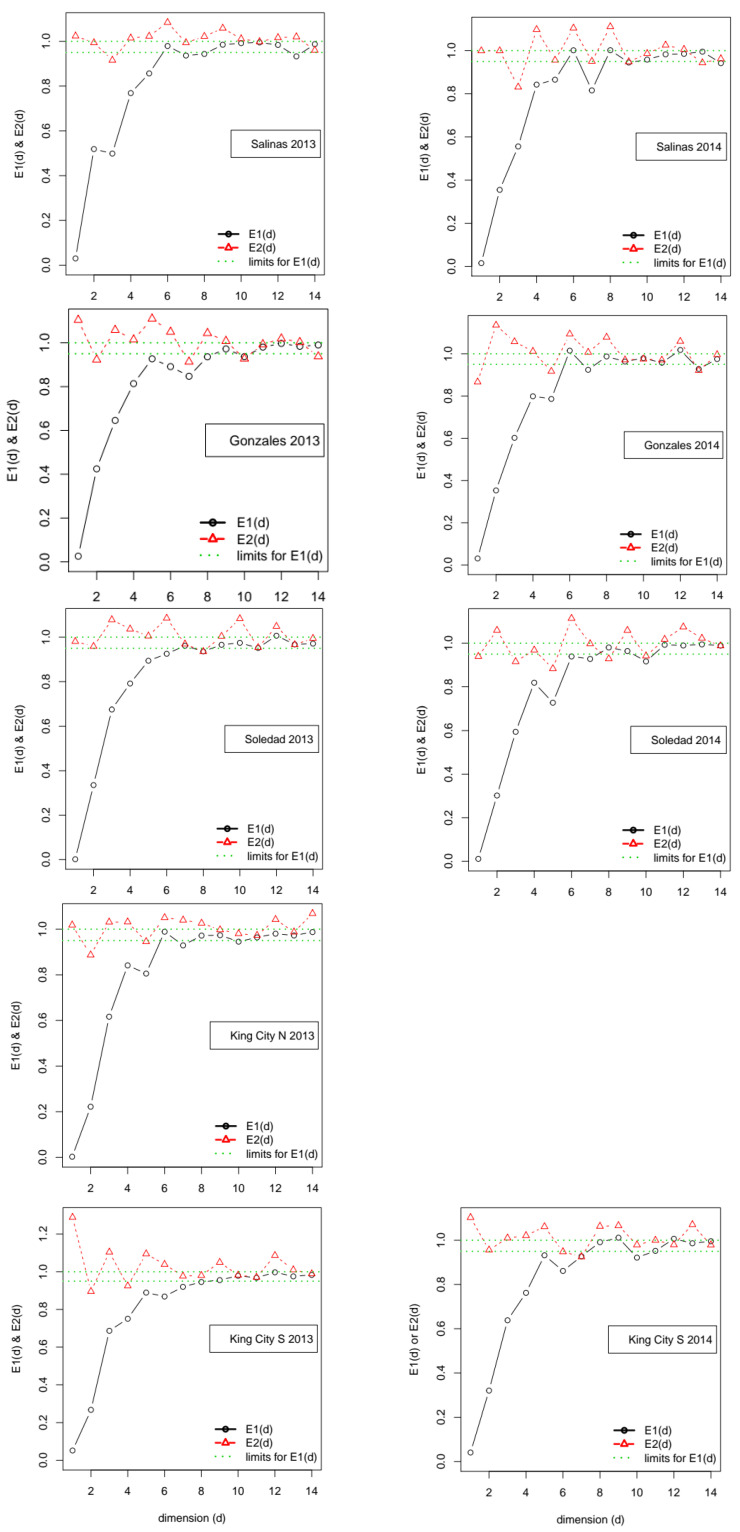
Output from the R function *estimateEmbeddingDim* for each series: the dimension, d, at which function E1 first falls within the critical region around 1 is taken to be the embedding dimension. The behavior of function E2 indicates the nature of the series. E2 is approximately equal to 1 for all values of d for all series, indicating that the series are stochastic in nature. Note that the embedding dimension is denoted with the letter *m* in the main text of the paper.

The results of using Cao’s [24] method to test for deterministic versus stochastic dynamics indicated that all 9 series had a stochastic nature; the value of function E2(*m*) stayed close to the value 1 for all values of *m* tested. Graphical output from the R function is given in Appendix A in Figure A1. Note that the R function uses the symbol *d* in the place of *m*.

**Figure 3:**
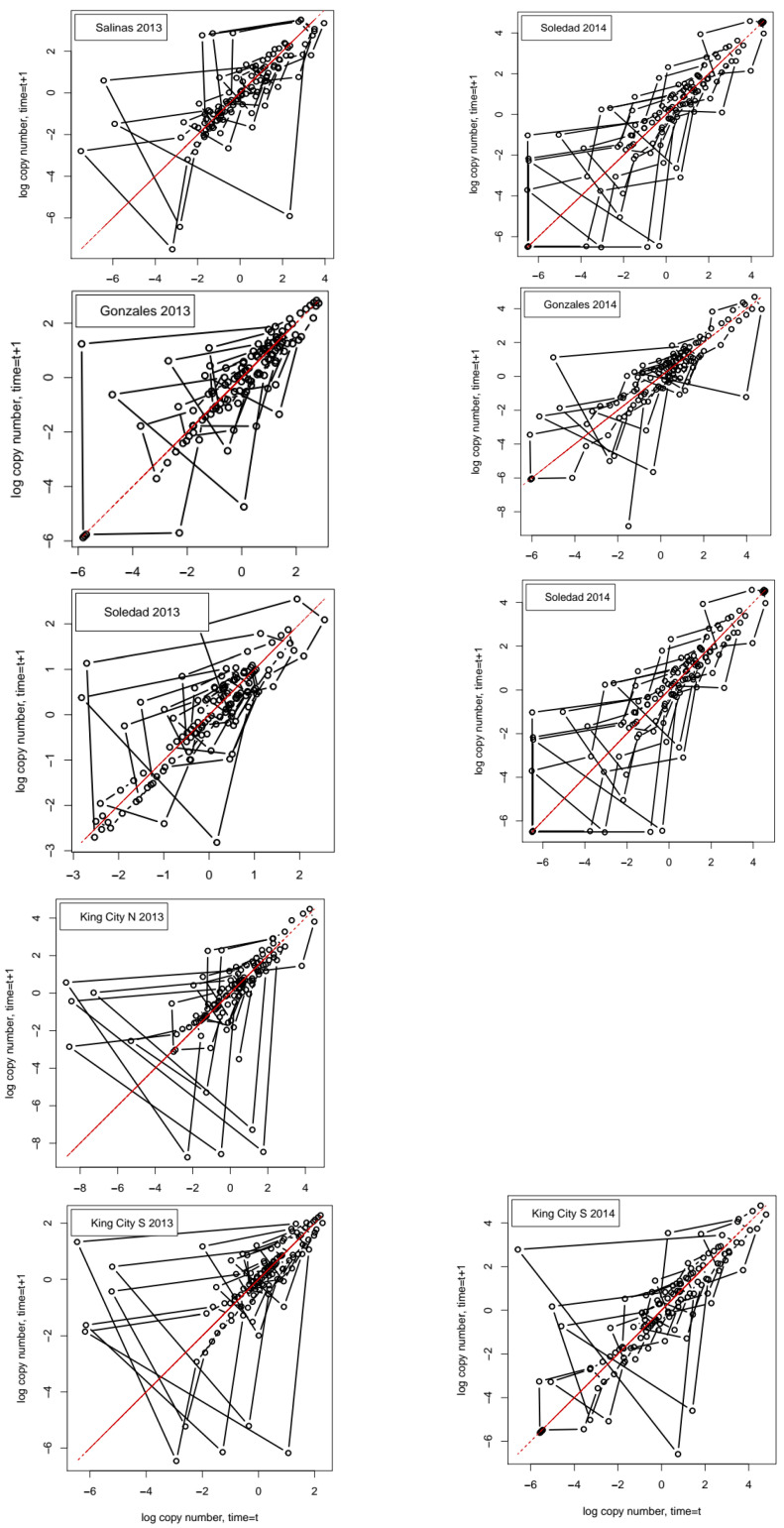
Phase space plots for the 9 series of detrended daily pathogen DNA copy numbers: the data are log*_e_* values of the detrended data. Series orbiting a fixed attractor or a limit cycle show clockwise orbits. The obvious tendency for the phase portraits to lie along the diagonal for which *n_t_* = *n_t_*_+1_ is partly an artifact of detrending and partly a result of the fact that the series all contain sequences of observations that are very close to the mean value of the detrended series.

The phase plots (Figure 3) for the detrended series show a strong tendency for the points to lie along the diagonal on which *n_t_* = *n_t_*_+1_, with short orbits away from this line, typically lasting no more than three to four time steps. These features are indicative of stochastic variation around a fixed value with a mixture of immediate and time-delayed feedback Turchin [26].

For all 9 series, the value of the dominant Lyapunov exponent (*λ*_1_) was greater than 0, indicating that chaotic divergence would occur in independent realizations generated by the same data generating process. Although positive, the values of *λ*_1_ were small, ranging from 0.04 to 0.17 (Table 1). Across the nine series, the value of the Lyapunov exponent was negatively correlated with the percent variance accounted for in fitting linear regression models to the series of instantaneous log growth rates and the coefficient of prediction for the linear fits; series with a higher Lyapunov exponent gave rise to poorer linear autoregressive models.

The best fitting autoregressive models for the time series of instantaneous rates generally captured only a low proportion of the variance in the series (Table 1). In general, the fitted values from the models showed less variability than the observed data, although in some cases, the qualitative fit to the series, in tracking the direction of the oscillations, was reasonably good. The main result from these analyses was that, while the data neither exhibited oscillations that could be easily attributed to a low-dimensional nonlinear attractor nor were they easily described by autoregressive linear models. The fitted autoregressive models and series of *R_t_* for each location/year combination are shown in Figure A3 in Appendix A.

The AMI and ACF functions were correlated, but there was no consistent tendency for the AMI to reach its first minimum at higher lag than ACF. The AMI function minimized at higher lag than the ACF in 6 of the 9 cases; the functions minimized at the same lag in one case; and in the remaining two cases, the AMI minimized at lower lag than the ACF. In general, the estimated embedding dimension, *m*, was similar to the value suggested by the first minimum of the AMI and ACF functions; across the nine series, *m* was negatively correlated with both AMI and ACF. The relatively large estimated values for *m* are indicative of complex dynamics in the observed data, but we note, again, that the data series are relatively short, which may affect the accuracy of the estimated parameter.

### 3.2. Entropy Measures of Time Series Uncertainty

We calculated entropy values along the time series for each location/year combination in two different ways. For the detrended copy number data, the entropy was calculated (in nats) using an automated binning procedure. The resulting series of entropy values are shown together with the data in Figure 1.

In the second year of observations (2014), the detection of pathogen DNA on the traps was sporadic. All four series showed an early peak in copy numbers around day 10 and then a long period of low-to-no detection until around day 80, when all locations experienced another peak in detection. Apart from these two shared features, the time series of trap counts were superficially dissimilar across the four locations sampled in 2014, but the series of cumulative entropy values showed a similar pattern in all four cases, with an initial peak corresponding to the trap data at day 10 followed by a long reduction as successive, similar trap results resulted in a reduction in heterogeneity in the data. The peak in trap counts caused a further peak in entropy around day 80, followed by a second period of decline. In general, the cumulative entropies in 2014 did not exceed 1.5 nats except in the case of King City, South, for which the initial peak was 1.78 nats. The final values for the entropy of the four series in 2014 are given in Table 1 and range from 0.88 to 1.14 nats.

In contrast to the more or less consistent pattern revealed by the 2014 data, the cumulative entropy values for the 2013 data sets were more variable. The final values for the five series tended to be higher than those in 2014 ranging from 1.00 to 2.04 nats with the exception of the King City North location, which had a final entropy value of 0.63 nats. In Salinas and Gonzales, the entropy value peaked early at over 2 nats and declined somewhat over the course of the season, although still finishing at or above 1.00 nats. In contrast to this early peak and decline pattern, at the remaining three sites in 2013, uncertainty increased through much of the season, in association with repeated oscillations in the trap copy number data.

**Figure 4:**
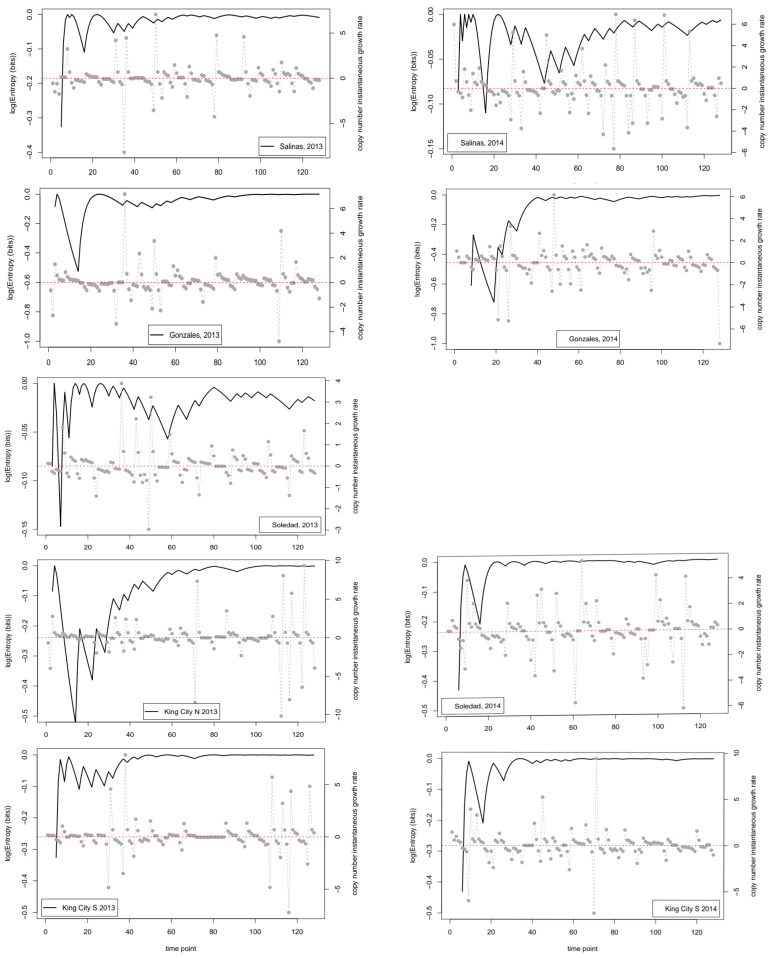
Graphs for 9 location years showing the series of instantaneous growth rates between successive time points (right axis scale) and the cumulative entropy (bits) of the series of binary values indicating whether the growth rate series is positive (left axis scale): the left axis is shown on a log*_e_* scale to allow the variation in the entropy values to be visible. Note that, on this scale, the theoretical maximum value is 0.

In addition to characterizing uncertainty in the daily trap data directly, we also assessed the uncertainty in the simpler issue of whether the observed series increased between each successive pair of days. Figure 4 shows the time series for the entropy of the cumulative binary series together with the corresponding series of *R_t_*, the instantaneous change in the log copy numbers between pairs of observations. The analysis showed that, in all nine series, the entropy remained close to its theoretical maximum value (i.e., 1 bit) over much of the season following an initial transient period lasting approximately 30 days. In three of the series (King City, North 2013; Soledad, 2013; and Salinas, 2014), the entropy did not settle close to its maximum until later in the season, but even in these cases, the final entropy value was close to the theoretical maximum of 1 bit. Note that, in Figure 4, the entropy values are shown on a log scale to allow detail of the changes over time to be visible.

## 4. Discussion

The quotation from the late Sir Robert May’s introduction to the Landmark edition [1] of his monograph *Stability and Complexity in Model Ecosystems* was chosen deliberately and for more than one reason. First, May’s point that the dynamics of real systems are likely to be a mixture of stochastic and deterministic processes applies directly to our observations on the time series of spore trap DNA copy numbers for *P. effusa* in the Salinas Valley of California. Secondly, May was an advocate of the idea that models can and should be used in biology in a strategic way to try to understand broad types of behavior without necessarily considering immediate questions of application or numerical accuracy in any specific case, while our analyses are predominantly statistical in nature, they are nonetheless carried out from a strategic perspective. Our aim in this study was not so much to produce accurate predictive models of any of the series as it was to use the tools of nonlinear time series analysis, together with some linear methods, to investigate the broad properties of pathogen DNA copy data collected from vortex air samplers.

**Figure A2:**
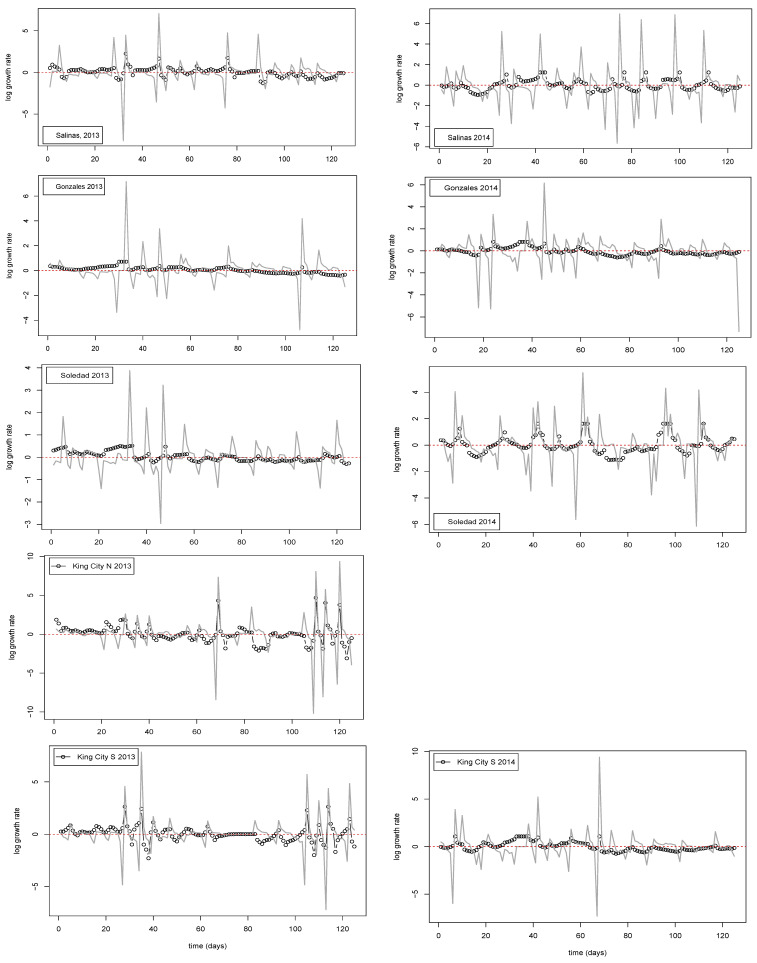
Correlation matrix plot indicating the numerical value and direction of correlations among the summary variables for time series properties across the 9 example times series. Abbreviations: ACF, autocorrelation function; PACF, partial autocorrelation function; AMI, average mutual information; m estimated embedding dimension; ly1, dominant Lyapunov exponent; Enctopy_CN, entropy (nats) for the pathogen DNA copy number; Entropy_bin, entropy (bits) for the binary series indicating whether first differences in trap copy numbers are positive; VAF, adjusted percentage variance accounting for (adjusted R2) the autoregressive linear model; Coeff_pred, prediction coefficient comparing the autoregressive model with the mean of the series used as a simple predictor.

**Figure A3:**
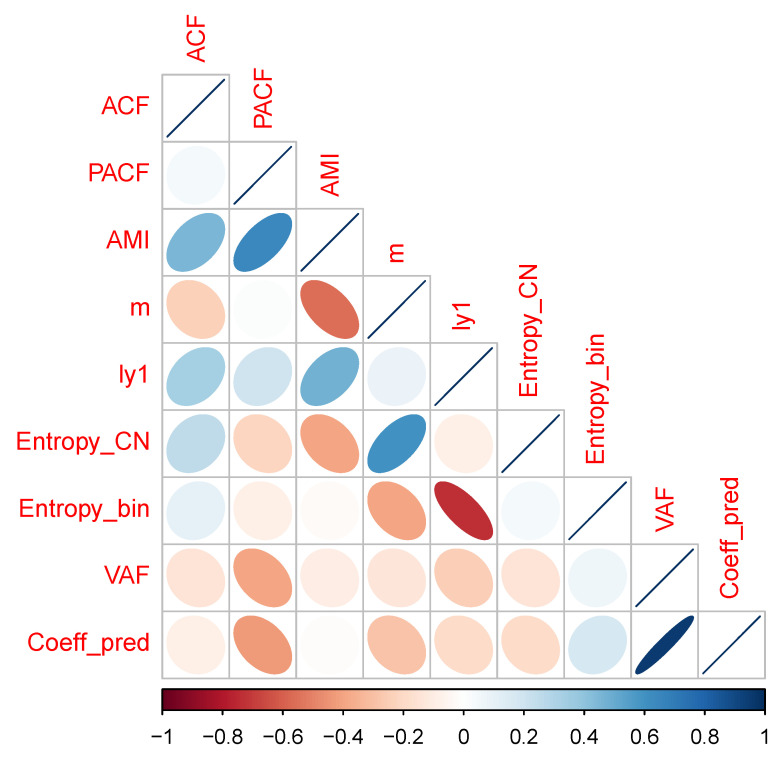
Observed data and fitted values for linear autoregressive models fitted to Equation (1) in the main text in the case of each of the nine series. Observed data: gray dashed line with open symbols; fitted values: black open symbols. The simple autoregressive models are capable of capturing some of the dynamic behavior in the series but generally lack the amplitude of the observed data and are poor at representing abrupt changes from large positive to large negative growth rates.

A correlation matrix plot for the numerical data in Table 1 is given as Figure A2 in Appendix A. Summarizing the results for the diagnosis of time series properties, a mixture of findings resulted. In some cases, there were indications of deterministic chaos—i.e., positive estimated values for the Lyapunov coefficient, failure of time reversibility test in surrogates—while others were indicative of stochastic noise, i.e., in 5 out of 9 cases, the surrogate test failed to reject the hypothesis of time reversibility, and the first minimum values of the ACF and AMI functions were generally similar, indicating that the more general information-theoretic test of association based on average mutual information did not detect dependence in the series beyond the linear association measured by the ACF.

All of the series had positive Lyapunov exponents, indicating a tendency for deterministic sensitivity to initial conditions [26]. On the other hand, application of Cao’s approach [24] indicated that the series were stochastic. The surrogate (bootstrap) test of time reversibility indicated that five series were compatible with the hypothesis that they were generated by a stochastic linear process while four were not. Relatively low values for the coefficient of prediction and adjusted-R^2^ calculated from linear autoregressive models, ranging from a minimum of 3.6% (Gonzales, 2013) to a maximum of 26.4% (King City, N, 2013), also suggested that the series were strongly influenced by stochastic noise. Taken together, these results indicated that the series lie in the transition between stochastic and deterministic uncertainty in what Turchin [26] refers to as *quasi-chaotic* territory at the boundary between the two types of dynamics.

It seems reasonable, based on the dependence of oomycete pathogens such as *P. effusa* on suitable weather for spore production and release, that the copy number on air sampler traps would show appreciable stochasticity. Not only is the number of DNA copies detected dependent on the response of the pathogen to uncertain weather conditions, the physical processes of dispersal, and transport in air, together with the vortex sampling process itself, meaning that there are multiple sources of stochasticity between the release of spores and subsequent trapping events. However, at the same time, crop management practices such as planting and harvesting salad spinach happen on cycles of between 21 and 45 days, and may be a source of deterministic forcing in the data complicating the dynamics. If the data are predominantly stochastic in nature, then traditional statistical models should be able to describe the pattern and to characterize the uncertainty. Similarly, Turchin [26] argues that dynamic patterns generated by low-dimensional attractors can also successfully be described by relatively simple models. Our analyses indicate that, at least in the case of *P. effusa* in the Salinas Valley in California, the observed dynamics may fall between these two preferred situations, making characterization of the dynamics difficult and leading to low overall predictability. The estimated embedding dimension for the series (after detrending) ranged from 6 to 9, indicating that they did not have dynamics compatible with a low-dimensional attractor.

If we consider the data in relation to variability in time and space, there are clear implications for making robust inferences about the quantity of pathogen inoculum in the air. For example, at three of the four locations where samplers were deployed in two successive years, the dynamics were classed as linear in one year and not linear in the other. The four locations sampled span a linear distance of approximately 80 km from Salinas in the north to King City in the south. In 2014, a year with relatively little pathogen activity, peaks in trap counts, and corresponding time series of entropy values showed relatively good agreement. In contrast, in 2013, when inoculum pressure was higher, generally, there was much less agreement between locations, and extrapolation from one location to another would not necessarily have yielded robust conclusions about the dynamics of the pathogen. The most striking example is the contrast between Salinas and King City S. In Salinas between day 20 and day 80, trap catches were relatively low and the cumulative value of the entropy showed a steady decline from approximately 1.5 nats to under 1 nat. In contrast, over the same period in King City, multiple peaks in trap catches were noted and entropy in the catch data rose from approximately 1 nat to approximately 2 nats.

Inevitably, in a first use of a new methodology in a specific field of application, there are numerous things that could have been done that were not. The focus of our analyses was on the dynamics and properties of the times series when analyzed on a daily time step. It is probably not surprising that the binary series indicating the direction of change was close to its maximum entropy value over much of the season in both years at most locations. This result suggests that, on average on any given day, a sample from the next day is as likely to be higher as it is to be lower than the sample from the current day. Technical advances in sample preparation are reducing the time it takes to process nucleic acid samples from spore traps. As a consequence, the apparent possibility of real-time forecasting of disease risk on a daily basis is increasing. One possible interpretation of our findings is, however, that the usefulness of such forecasts may be limited by the inherent uncertainty of the data. Is there predictive value for decision making in knowing today’s trap value if tomorrow’s value may be higher or lower with equal probability? The results obtained here indicate that the binary strings derived from the time series data are close to being simple sequences of independent Bernoulli trials with a probability of 0.5 determining the outcome. As Grünwald [27] points out, model selection and fitting can be considered as analogous to data compression, and when a string of bits is essentially random, it is difficult to achieve an accurate description (i.e., compression) of the data that is more concise than simply writing the data out.

Aggregating results to form moving averages over longer runs of days would perhaps lead to information values that were more easily linked to disease outcomes, but the detailed work to examine this issue lies beyond the scope of this study. The objective of our work was not to explore whether entropy values can be used as a predictive indicator for disease risk but to characterize the uncertainty of time series data from spore traps to give practitioners a richer perspective on the level and nature of the uncertainty inherent in the data they collect.

Looking at the entropy values for the two seasons retrospectively, it is clear (Figure 1) that the cumulative entropy values have a qualitatively different nature in the two seasons. Moving from consideration of uncertainty within a season to differences in uncertainty between seasons, the results presented here suggest that information quantities might provide an alternative means of classifying growing seasons, but the extra value to epidemiology (compared with what can be learned from direct comparison of the trap data themselves, for example) that might be gained from long-term comparative analyses is not known at this time. We hope others will be encouraged to further analyze the open questions raised here; this is a new field for future research.

Our analyses suggest that there may be quite severe practical limitations to being able to characterize pathogen dynamics using the combination of vortex air sampling and DNA target amplification. There are clear cases where detection of primary inoculum helps to improve disease management [9,11], but the situation regarding season-long disease management based on measuring secondary inoculum is much less clear. There are few, if any, other published datasets for comparison so the potential remains uncertain. However, even with data series extending to over 100 data points, the fact that the coefficient of prediction for autoregressive models was close to zero is an indication that the time series may be so noisy that extracting a useful, succinct model of the dynamics may be difficult. While our results point to restrictions in the utility of dynamical analysis for helping with practical problems in disease risk forecasting, at the same time, they suggest a great deal of interesting investigative research on inoculum dynamics and their positions on the continuum form pure deterministic complexity to pure stochastic noise.

## Figures and Tables

**Table 1:** Summary statistics for the 9 time series of pathogen DNA copy number.

	Sal13	Sal14	Gon13	Gon14	Sol13	Sol14	KcN13	KcS13	KcS14 ^2^
ACF ^1^	3	6	9	7	5	8	5	7	5
PACF	4	8	3	10	6	5	4	5	5
AMI	5	11	6	10	4	10	6	7	6
*m*	6	6	6	6	9	6	6	7	7
*λ*_1_	0.05	0.16	0.09	0.06	0.13	0.18	0.04	0.04	0.05
Linear?	N	Y	Y	N	N	Y	N	Y	Y
Entropy, nats (copy no.)	1.00	0.98	1.58	0.88	1.70	0.88	0.63	2.04	1.14
Entropy, bits (binary)	0.99	0.99	1.00	1.00	0.98	0.98	1.00	1.00	1.00
%VAF	11.4	6.6	3.6	4.2	7.1	17.0	26.4	20.1	7.5
pred Coeff	0.14	0.14	0.10	0.06	0.08	0.18	0.30	0.22	0.09

^1^ ACF, lag at which series autocorrelation function has first minimum; PACF, lag at which the partial autocorrelation function has its first minimum; AMI, lag at which the series average mutual information function has its first minimum; *m*, estimated embedding dimension; *λ*_1_, the maximum Lyapunov exponent; Linear?, outcome of surrogate test for compatibility of the series with stochastic linearity; Entropy copy no., estimated entropy (nats) of the copy number time series; Entropy binary, entropy (bits) of the binary series indicating if the copy number increased between successive pairs of observations; %VAF, percent variance accounted for in the best autoregressive linear model for the series of instantaneous rates of change in the log copy number data; pred Coeff, prediction coefficient for the autoregressive linear model (see text for details). ^2^ Location/year combination: Sal, Salinas; Gon, Gonzales; Sol, Soledad; KcN, King City, North; KcS, King City, South; 13, 2013; 14, 2014.

## Supplementary Materials

The following are available online at https://github.com/robchoudhury/spore_trap_information_theory, R code to reproduce results for Salinas, 2013:Sal_13.R, Data required to run the analysis for Salinas 2013: spore_fill.csv, R Project file:spore_trap_information_theory.Rproj.

## References

[1] May R.M. (2019). Stability and Complexity in Model Ecosystems.

[2] Madden L.V., Hughes G., Van Den Bosch F. (2007). The Study of Plant Disease Epidemics.

[3] Zwankhuizen M., Zadoks J. (2002). *Phytophthora infestans*’s 10-year truce with Holland: A long-term analysis of potato late-blight epidemics in the Netherlands. Plant Pathol..

[4] Kriss A., Paul P., Madden L. (2010). Relationship between yearly fluctuations in Fusarium head blight intensity and environmental variables: A window-pane analysis. Phytopathology.

[5] Carisse O., McRoberts N., Brodeur L. (2008). Comparison of monitoring-and weather-based risk indicators of botrytis leaf blight of onion and determination of action thresholds. Can. J. Plant Pathol..

[6] Choudhury R., Koike S., Fox A., Anchieta A., Subbarao K., Klosterman S., McRoberts N. (2016). Season-long dynamics of spinach downy mildew determined by spore trapping and disease incidence. Phytopathology.

[7] Kasprzyk I., Worek M. (2006). Airborne fungal spores in urban and rural environments in Poland. Aerobiologia.

[8] Klosterman S.J., Anchieta A., McRoberts N., Koike S.T., Subbarao K.V., Voglmayr H., Choi Y.-J., Thines M., Martin F.N. (2014). Coupling spore traps and quantitative PCR assays for detection of the downy mildew pathogens of spinach (*Peronospora effusa*) and beet (*P. schachtii*). Phytopathology.

[9] Carisse O., Tremblay D., Lévesque C., Gindro K., Ward P., Houde A. (2009). Development of a TaqMan real-time PCR assay for quantification of airborne conidia of Botrytis squamosa and management of Botrytis leaf blight of onion. Phytopathology.

[10] Carisse O., Tremblay D., McDonald M., Brodeur L., McRoberts N. (2011). Management of Botrytis leaf blight of onion: The Québec experience of 20 years of continual improvement. Plant Dis..

[11] Falacy J., Grove G., Mahaffee W., Galloway H., Glawe D., Larsen R., Vandemark G. (2007). Detection of *Erysiphe necator* in air samples using the polymerase chain reaction and species-specific primers. Phytopathology.

[12] Huffaker R., Bittelli M., Rosa R. (2017). Nonlinear Time Series Analysis with R.

[13] Kantz H., Schreiber T. (2004). Nonlinear Time Series Analysis.

[14] Garcia C., Sawitzki G. (2020). Nonlineartseries: Nonlinear Time Series Analysis. https://cran.r-project.org/web/packages/nonlinearTseries/index.html.

[15] Fabio Di Narzo A. (2019). TseriesChaos: Analysis of Nonlinear Time Series. https://cran.r-project.org/web/packages/tseriesChaos/tseriesChaos.pdf.

[16] Giannerini S. (2017). TseriesEntropy: Entropy Based Analysis and Tests for Time Series. https://cran.r-project.org/web/packages/tseriesEntropy/tseriesEntropy.pdf.

[17] Hausser J., Strimmer K. (2015). Entropy: Estimation of Entropy, Mutual Information and Related Quantities. https://cran.r-project.org/web/packages/entropy/entropy.pdf.

[18] Granger C.W., Maasoumi E., Racine J. (2004). A dependence metric for possibly nonlinear processes. J. Time Ser. Anal..

[19] Takens F. (1981). Detecting strange attractors in turbulence. Dynamical Systems and Turbulence.

[20] Theiler J. (1986). Spurious dimension from correlation algorithms applied to limited time series data. Phys. Rev. A.

[21] Theiler J. (1990). Estimating fractal dimension. JOSA A.

[22] Provenzale A., Smith L.A., Vio R., Murante G. (1992). Distinguishing between low-dimensional dynamics and randomness in measured time series. Phys. D Nonlinear Phenom..

[23] Casdagli M., Eubank S., Farmer J., Gibson J., Desjardins D., Hunter N., Theiler J. Nonlinear modeling of chaotic time series: Theory and applications. Proceedings of the Electric Power Research Institute (EPRI) Workshop on Applications of Chaos.

[24] Cao L. (1997). Practical method for determining the minimum embedding dimension of a scalar time series. Phys. D Nonlinear Phenom..

[25] Royama T. (2012). Analytical Population Dynamics.

[26] Turchin P. (2003). Complex. Population Dynamics: A Theoretical/Empirical Synthesis.

[27] Grünwald P.G. (2007). The Minimum Description Length Principle.

